# Abundant DNA 6mA methylation during early embryogenesis of zebrafish and pig

**DOI:** 10.1038/ncomms13052

**Published:** 2016-10-07

**Authors:** Jianzhao Liu, Yuanxiang Zhu, Guan-Zheng Luo, Xinxia Wang, Yanan Yue, Xiaona Wang, Xin Zong, Kai Chen, Hang Yin, Ye Fu, Dali Han, Yizhen Wang, Dahua Chen, Chuan He

**Affiliations:** 1 Department of Chemistry, Department of Biochemistry and Molecular Biology, Institute for Biophysical Dynamics, Howard Hughes Medical Institute, The University of Chicago, 929 East 57th Street, Chicago, Illinois 60637, USA; 2 State Key Laboratory of Reproductive Biology, Institute of Zoology, Chinese Academy of Sciences, Beijing 100101, China; 3 Key Laboratory of Animal Nutrition and Feed Sciences, Ministry of Agriculture, College of Animal Sciences, Zhejiang University, No. 866 Yuhangtang Road, Hangzhou, Zhejiang 310058, China

## Abstract

DNA *N*^6^-methyldeoxyadenosine (6mA) is a well-known prokaryotic DNA modification that has been shown to exist and play epigenetic roles in eukaryotic DNA. Here we report that 6mA accumulates up to ∼0.1–0.2% of total deoxyadenosine during early embryogenesis of vertebrates, but diminishes to the background level with the progression of the embryo development. During this process a large fraction of 6mAs locate in repetitive regions of the genome.

## Abstract (web summary)

DNA 6mA is a poorly understood epigenetic mark present at a low abundance in eukaryotic genomes. Here the authors observe high levels in zebrafish and pig during early embryogenesis enriched to repetitive regions of the genome and followed by attenuation during development.

## Graphical abstract

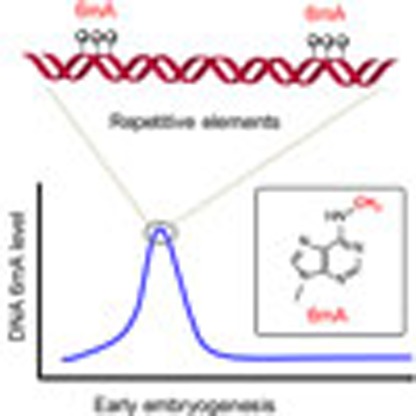

Dynamic DNA methylations expand the genetic code beyond the four canonical bases and carry important epigenetic information that can be transmitted among generations of cells or organisms^1^. DNA methylations such as *N*^4^-methyldeoxylcytosine, 5-methyldeoxycytosine (5mC or m^5^dC) and *N*^6^-methyldeoxyadenosine (6mA or m^6^dA) are known to exist and carry functional roles in living organisms^2^. *N*^4^-methyldeoxylcytosine and 6mA are primarily used by prokaryotes in the restriction-modification system to protect their own genomes from foreign DNA invasion^2^, whereas 5mC is best known for its significant epigenetic roles in eukaryotes, especically in mammals and plants^3^,^4^.

Although both 6mA and 5mC were discovered almost at the same time a few decades ago, attention to 6mA in eukaryotes has been limited mostly due to its low abundance in multicellular eukaryotes and technological limitations. In most cells and tissues of vetebrates including mammals, 6mA is either detected at a few to tens p.p.m. (part per million A) level or simply not detectable at all using modern mass spectrometry technique^5^. The extremely low abundance of 6mA in most organisms, at a level similar to DNA base damage, has cast in the past further doubt about the functional relevance of 6mA. Very recently, we and others reported the discovery/characterization of 6mA in *Chlamydomonas reinhardtii*^6^, *Caenorhabditis elegans*^7^ and *Drosophila*^8^, suggesting its potential epigenetic functions^9^,^10^. In *C. reinhardtii*, 6mA was found to be located in the AT motif near the transcription start sites. It marks active genes and is associated with nucleosome positioning^6^. In the genomes of *C. elegans* and *Drosophila*, 6mA affects *trans*-generational inheritance^7^ and expression of certain transposons^8^, respectively. Although we have been working on 6mA in vertebrates and mammals, a recent publication has reported the detection of 6mA in *Xenopus laevis* and mammals, albeit with very low levels of 6mA observed (0.00009%)^11^. A more recent work reported a potential 6mA demethylase in mammals, which still needs to be validated with biochemical and physiological studies^12^. The 6mA level reported is again quite low even after enrichment. This modification was found to be depleted in coding regions and might exist in the AG sequence content. In this work, we characterize DNA 6mA modification in zebrafish and pig. We found that 6mA accumulates to relatively high abundances during early embryogenesis (6mA/A up to ∼0.1–0.2%), but is attenuated to the background p.p.m. level with the development progression. During this process, a substantial set of 6mAs locate in repetitive regions of the genome.

## Results

### DNA 6mA modification during early embryogenesis of zebrafish

We have been searching biological processes during which 6mA accumulates to relatively high abundance. We reason that an abundant accumulation and a dynamic change of 6mA could indicate functional relevance. After quantifying 6mA levels in various cell types and tissues, we noticed that 6mA accumulates to surprisingly high levels during early embryogenesis in vertebrates such as zebrafish and pig. Using zebrafish as a model, we purified and quantified genomic DNAs (gDNAs) from sperm, oocyte, early embryos with different time point and adult fish organs by using a sensitive ultra-high-performance liquid chromatography coupled with a triple-quadrupole tandem mass spectrometry (UHPLC–QQQ–MS/MS) assay with pure 6mA nucleoside as an external standard^6^. We devoted significant efforts to the collection of the oocytes and early embryos, to purify sufficient gDNA for LC–MS/MS measurements. On average, we collected over 10,000 cells at each embryo stage and purified gDNA.

**Figure 1:**
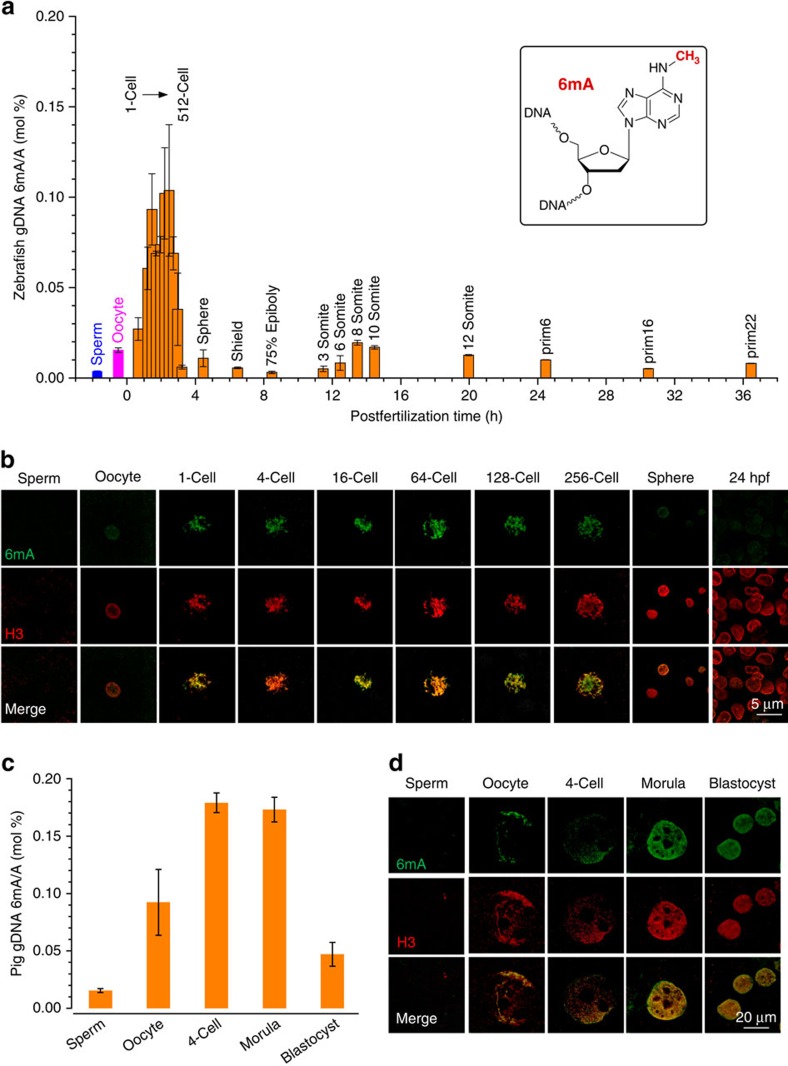
6mA modification in gDNAs of gametes and selected early embryos in both zebrafish and pig (**a**,**c**) Quantification of 6mA modification in isolated gDNA from sperm, oocyte and various embryo stages of zebrafish (**a**) and pig (**c**) by UHPLC–QQQ–MS/MS. The mole ratios of 6mA/A are shown, error bars indicate mean±s.d. (*n*=3). (**b**,**d**) Immunofluorescence images of selected embryo stages of zebrafish (**b**) and pig (**d**) at single-cell level stained by anti-6mA antibody (green, rabbit polyclonal from SYSY) and anti-Histone 3 (H3) antibody (red, mouse monoclonal from Biodragon Immunotechnologies). Early embryo stages show strong fluorescence indicative of the high abundance of 6mA in gDNA, whereas the signal is diminished with the progression of the embryo development. Full embryo images are presented in Supplementary Fig. 3.

The LC–MS/MS result shows that the sperm gDNA contains around 0.003% of 6mA/A, whereas the oocyte contains fivefold higher 6mA than in sperm. On zygote formation, the level of 6mA increases and doubles that of oocyte. Interestingly, starting from the 1-cell zygote, the level of 6mA in DNA further increases along with development and reaches a maximum of ∼0.1% 6mA/A at around 32-cell to 64-cell embryo stage corresponding to ∼2 h post fertilization (2 hpf; Fig. 1a and Supplementary Fig. 1a)^13^. Afterwards, the 6mA level gradually decreases to around 0.006% at 512-cell stage and stays at this low level till prim22 (∼36 hpf). We further characterized adult fish organs including the brain, eye, heart, ovary, testis, muscle and intestine; all of them yielded a relatively low ratio of 6mA/A at 0.002−0.004% (Supplementary Fig. 1b). Dot blot assay was used as an independent way to measure 6mA levels at selected zebrafish early development stages (Supplementary Fig. 2), showing results consistent with LC–MS/MS experiments.

### Immunostaining of 6mA in zebrafish embryonic gDNAs

To exclude the possibility that the detected LC–MS/MS signal is due to foreign 6mA contamination, we applied immunofluorescence staining of the embryos^14^ with anti-6mA-specific antibody (after treating with RNase A to digest and wash away all RNAs) for 6mA detection in gDNA. Very weak fluorescence was observed from the sperm staining, while oocyte staining displayed stronger signals (Fig. 1b and Supplementary Fig. 3a). Starting from the zygote stage, the nuclear immunofluorescence of embryos increases significantly along with the development, reaching its maximum at about 32-cell to 64-cell stage (Fig. 1b). Afterwards, the signal decreases with the progression of the development. These observations are consistent with the LC–MS/MS result. After treating with DNase, the immunofluorescence signal disappeared (Supplementary Fig. 3b). Together, LC–MS/MS, dot blot assay and immunostaining results confirm the existence of abundant 6mA during early embryogenesis of zebrafish. Mammalian cells and tissues tend to contain a few percent of 5mC and 0.01−1% 5-hydroxymethycytosine (5hmC) in gDNA. Our observation of 6mA during early embryogenesis represents the first example that an abundant 6mA level comparable to those of 5mC and 5hmC is observed in vertebrates. The dynamic changes suggest functional roles of 6mA during the process. To further substantiate this observation in mammals, we studied early embryogenesis in pig.

### Quantification of DNA 6mA level in pig embryos

It is much harder to obtain sufficient pig oocytes and embryos. The current available way to obtain early embyros is done through *in vitro* fertilization followed by culturing into different embryonic stages^15^; however, this method is restricted by low fertilization rates. Owing to the technical limitation, the successfully fertilized oocyte can only be cultured to, at most, the blastocyst stage. To have enough gDNA for LC–MS/MS and immuostaining experiments, we devoted significant efforts to collecting sufficient materials. Besides pig sperm and oocyte, we selected 4-cell, morula and blastocyst stages (∼2,500 oocytes, ∼1,800 4C stage, ∼900 morulae and ∼500 blastocysts) and purified gDNAs for LC–MS/MS measurement (Fig. 1c). Similar to zebrafish, the 6mA/A ratio in oocyte (0.09%) is approximately six times higher than that of sperm. This ratio rises to ∼0.17% from the four-cell to the morula stage and then decreases to 0.05% at the blastocyst stage. The corresponding immunostaining results are consistent with LC–MS/MS measurements (Fig. 1d and Supplementary Fig. 3c), which further supports the presence of abundant 6mA. We also checked the 6mA level in the gDNAs of various adult pig tissues with only low p.p.m. level observed (Supplementary Fig. 1c).

### Distribution of 6mA in zebrafish embryonic gDNAs

Next, we studied the genomic distribution of 6mA in selected systems. The antibody-based (SYSY 6mA antibody) immunoprecipitation of 6mA-containing DNA followed by next-generation high-throughput sequencing (6mA-IP-seq)^6^ was used to map the genomic distribution patterns of 6mA. The 6mA-IP-seq generally requires micrograms of gDNA because of the unavoidable loss of materials in many purification steps during the construction of sequencing libraries. A large number of embryos would be required, to obtain sufficient gDNA materials, in particular at the early stages. We collected 5,000 zebrafish embryos at the 64-cell stage (∼1 μg gDNA isolated) in which 6mA accumulates to the highest level, as well as gDNAs from later stages including 11 hpf, 12 hpf and 13 hpf, and performed 6mA-IP-seq. Owing to the difficulty of acquiring enough gDNA from pig embyros, we were unable to sequence pig samples at the current stage.

**Figure 2:**
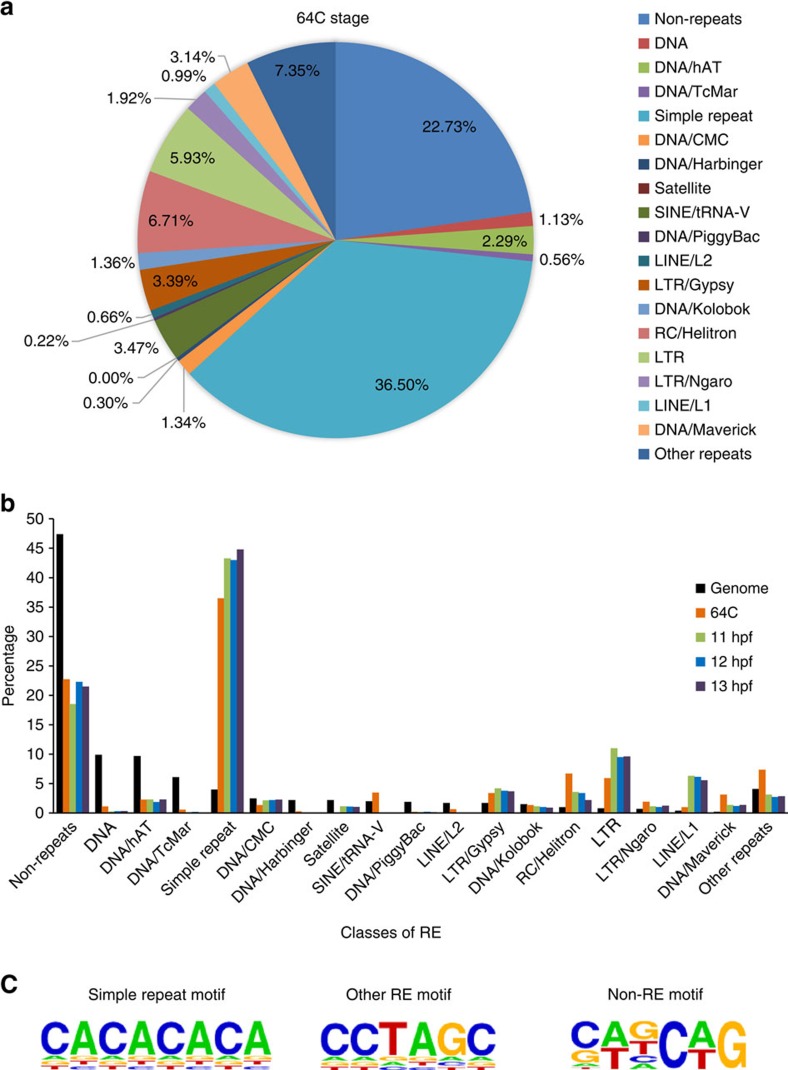
DNA 6mA-IP-seq reveals the distribution of 6mA across the genome of zebrafish at selected embryonic stages. (**a**) Pie chart showing the distribution of 6mA peaks at 64-cell (64C) stage. The peaks located in REs are classified into subgroups based on RepeatMasker annotation. (**b**) 6mA peak classification for samples of 64C, 11 hpf, 12 hpf and 13 hpf. The genome background distribution is shown for comparison. The peaks are classified with the same criteria shown in **a**. (**c**) Sequence motifs of 6mA peaks in simple repeat, other REs apart from simple repeats (other RE) and non-repetitive regions (non-RE) for the 64C stage sample. Motifs were searched and generated by Homer software. The *P*-values for simple repeat, RE and non-RE motifs are 5e−1163, 1e−1557 and 1e−390, respectively.

We performed bioinformatic analysis of the sequencing data generated from the zebrafish genome. By comparing 6mA-IP with input data, we identified ∼57,000 to ∼3,300 potential 6mA peaks in samples of 64C, 11 hpf, 12 hpf and 13 hpf (Supplementary Fig. 4a), among which the 64C samples possess the most peaks, consistent with the 6mA abundance measurements. These samples show peak overlapping ratio of 41−50% across different time points (Supplementary Fig. 4b). In general, the 6mA peaks are distributed widely across the genome with slight enrichment after transcription start site (TSS; Supplementary Fig. 5) and they were found to be enriched at exon, but not intron, intergenic nor promoter regions (Supplementary Fig. 6), which is different from the exon depletion case observed in the *X. laevis* testis genome^11^. Specifically, we found that ∼78–81% of the peaks locate in repetitive elements (REs), which suggests a close relationship between 6mA and RE (Fig. 2a and Supplementary Fig. 7a). Interestingly, 6mA peaks in the simple repeat region accounts for 37−45% of the total peaks. We further divided the RE regions into subtypes based on RepeatMasker^16^. Compared with the background constitution of RE regions in the genome, 6mA peaks enriches in simple repeat, RC/Helitron (rolling-circle transposon), LTR (long terminal repeat retrotransposon), LINE/L1 (long interspersed element) and DNA/Maverick (virus-like DNA transposon) classes of REs (Fig. 2b), thus reinforcing the finding that 6mA is correlated with REs. Sequencing of biological replicates at 11 hpf, 12 hpf and 13 hpf stages revealed good consistency (Supplementary Figs 4 and 7b, and Supplementary Table 1).

### Consensus motifs containing 6mA

To search for sequence preference of 6mA, we performed motif analysis on 6mA peak regions. Considering the sequence diversity between RE and non-RE regions, we divided peaks to three groups: simple repeat regions, other RE regions apart from simple repeats and regions not related to RE (non-RE). Interestingly, we obtained significant but also diverse motifs for each group. For instance, at 64C stage the regions of simple repeat, other RE regions and non-RE are mainly featured with 5′-CACACACA-3′, 5′-CCTAGC-3′ and 5′-CAGCAG-3′ motifs, respectively (Fig. 2c). Among the simple repeats, tandem CA motif is the most prevalent, which is followed by tandem 5′-TCCA-3′ and 5′-CCAA-3′ motifs. The most significant motif sequences showed recurrence in all sequencing samples (Supplementary Fig. 7c) and are different from those observed in a recent study^11^.

### 6mA-IP-seq by using different anti-6mA antibody

To avoid potential sequencing bias introduced by antibody, we chose another 6mA antibody (Abcam) to perfrom independent 6mA-IP-seq for the 64C stage and found 6mA distribution pattern and peak motif consistent with those described above by using the SYSY antibody (Supplementary Fig. 8). Simple repeat regions are again significantly enriched with 6mA, accounting for ∼53% peaks across the genome. These two antibodies show slightly different affinities to 6mA with the Abcam antibody possessing a relatively lower affinity but slightly higher selectivity (Supplementary Fig. 9); the use of the Abcam antibody appears to further enrich 6mA-abundant DNA segments. This result further suggests that simple repeat region is more enriched with 6mA modification.

### Selected 6mA-containing targets

To probe the correlation between the expression of REs enriched with 6mA and the timing of accumulation, we selected a few targets and used quantitative reverse transcription PCR to quantify their transcript expression levels at various developmental stages (Supplementary Fig. 10). Among the selected targets, *Thy1* (LTR/Gypsy), *Parp4* (LTR/Gypsy) and *Hel1* (RC/Helitron) all showed a trend of reduced expression with the progression of development. These data suggest that the expression of REs might be positively correlated with the 6mA abundance.

## Discussion

6mA is a well-studied prokaryotic DNA marker. In *Escherichia coli*, 6mA is present in the palindromic 5′-GATC-3′ motif and participates in the control of chromosome replication, nucleoid segregation, mismatch repair and transcription regulation^17^. 6mA is also present in eukaryotes and appears to have functional roles^10^. We show here that 6mA can accumulate to ∼0.1−0.2% of 6mA/A during early embryogenesis in zebrafish and pig. Levels of 6mA in oocyte are several folds higher than those of sperms (p.p.m. level) and, on zygote formation, the abundance of 6mA increases (6mA/A up to ∼0.1−0.2%) and then decreases (back to p.p.m. level) with the progression of embyronic development. 6mA prefers to be enriched in REs, whose expression might be positively correlated with the 6mA abundance. The highest abundance of 6mA observed appoaches the level of 5hmC in most mammalian tissues. Future studies should reveal how 6mA may correlate 5mC reprogramming in mammals^18^, the role of 6mA in REs^12^ and other regions (with motifs such as 5′-CACACACA-3′, 5′-CCTAGC-3′ and 5′-CAGCAG-3′), enzymes that install and/or erase 6mA and potential ‘reader' proteins that may bind 6mA to mediate biological functions.

## Methods

### Zebrafish strains and husbandry

Zebrafish (*Danio rerio*) embryos were derived from the Tubingen zebrafish lines. Embryos were incubated in Holtfreter's solution at 28.5 °C.

### Collection of early embryos and isolation of their gDNAs

Unfertilized sperm were squeezed out of anaesthetized males and oocytes were squeezed out of anaesthetized females (the oocytes were activated when they were squeezed into Holtfreter's solution; the polar bodies were extruded from the egg surface following egg activation). The fertilized embryos were grown in Holtfreter's solution at 28.5 °C and were staged according to standard morphological criteria. The oocytes and different stages of embryos were frozen in liquid nitrogen until enough embryos were collected for DNA extraction. For sperm, they were directly squeezed into lysis solution (100 mM Tris-HCl pH 8.5, 200 mM NaCl, 5 mM EDTA, 0.2% SDS and 50 μg ml^−1^ proteinase K) for gDNA extraction. For oocytes and early embryos, to each aliquot of 300 dechorionated embryos was added 600 μl of lysis solution. The solution was incubated in a rotation oven at 55 °C overnight. Equal volume of phenol/chloroform (pH 8.0) was added for extraction and the mixture was vortexed briefly and spun for 10 min in a microfuge at 16,000 *g*. To the collected aqueous solution 2 μl of RNase A (10 mg ml^−1^) was added and the resultant solution was incubated in thermomixer at 37 °C for additional 3 h. Phenol/chloroform extraction and DNA precipitation with 1/10 volume of 5 M NaCl and 1 volume of isopropanol were performed. The obtained DNA pellet was dissolved with 3dH_2_O.

### Pig oocyte collection and *in vitro* maturation

Ovaries of prepubertal gilts were collected in a local abattoir and transported to the laboratory at 37 °C in a 0.9% NaCl solution supplemented with kanamycin and penicillin at 30−35 °C within 3 h. Antral follicles of 3–6 mm were aspirated to collect the cumulus–oocyte complex (COCs) using a syringe. After washing three times with PVA-TL-HEPES media, the oocyte selection started with a screening for those oocytes that had a multilayered compact cumulus and a homogeneous ooplasm. The oocytes were washed twice again with TCM-199 media, which had been equilibrated for 3 h at 38.5 °C under 5% CO_2_. Subsequently, the COCs were matured in TCM-199 media without hormones at 38.5 °C in a humidified atmosphere of 5% CO_2_ for 42−44 h. Then, the COCs were washed in the working solution (0.03 g hyaluronidase, 5.46 g mannitol, 0.001 g BSA, 5 ml PVA-TL-HEPES and 95 ml ddH_2_O) to remove the cumulus cell and the mature oocytes were picked out for storage at −80 °C.

### *In vitro* fertilization

After maturation, the COCs were washed gently with 2 ml fresh IVF medium, to remove cumulus cells. After washing, denuded oocytes were placed in 50 μl drops of IVF medium covered with mineral oil. The oocytes were incubated at 38.5 °C in an atmosphere of 5% CO_2_ for 30 min until the spermatozoa were added. The sperms were washed twice by DPBS (136.89 mM NaCl, 2.68 mM KCl, 8.1 mM Na_2_HPO_4_ and 1.46 mM CaCl_2_.2H_2_O) at 12,000 *g* min^−1^ for 30 s and the resulting pellet was suspended in the IVF medium. After appropriate dilution, 50 μl of this sperm suspension was added to a 50 μl drop of IVF medium containing the oocytes at an oocytes:spermatozoa ratio of 1:4,000. The oocytes were incubated with the spermatozoa in IVF medium for 6 h at 38.5 °C under 5% CO_2_. Next, they were transferred into 500 μl PZM-3 medium and cultured for another 2 days to the 4-cells stage, 4 days to the morula stage and 7 days to the blastocyst stage, and the samples were stored at −80 °C for further assays.

### Mediums used in pig oocyte maturation and fertilization

The related medium recipes are shown as follows.

PVA-TL-HEPES: 6.663 g NaCl (Sigma-S-5886), 0.239 g KCl (Sigma, P4504), 0.168 g NaHCO_3_ (Sigma, S8875), 0.041 g NaH_2_PO_4_ (Sigma, S5011), 0.102 g MgCl_2_.6H_2_O (Sigma, M0250), 2.383 g HEPES (Sigma, H3784), 0.065 g penicillin (Sigma, P3032), 0.010 g Phenol Red (Sigma, 5,530), 0.294 g CaCl_2_.2H_2_O (Sigma,C7902), 0.1 g polyvinyl alcohol (PVA, Sigma, P8136), 2.186 g sorbitol (Sigma, S1876), 0.025 g Gentamicin (Gibco, 15710-064), 0.022 g sodium pyruvate (Sigma, P4562), 998 ml Milli Q H_2_O and 1.868 ml Na Lactate (Sigma, L7900), adjusting pH to 7.2−7.4 and osmotic pressure to 295−310 mOsm.

TCM-199: 3.05 mM D-glucose (Sigma, G7021), 0.91 mM sodium pyruvate (Sigma, P4562), 0.1% PVA (Sigma, P8136), 75 μg ml^−1^ penicillin (Sigma, P3032), 50 μg ml^−1^ streptomycin (Sigma, S1277), 0.5 μg ml^−1^ luteinizing hormone (Sigma, L5269), 10 ng ml^−1^ epidermal growth factor (Sigma, S4127), 0.5 μg ml^−1^ follicle stimulating hormone (Sigma, F2293), 0.57 mM cysteine (Sigma, C8152) and 10% porcine follicular fluid.

IVF medium (mTBM): 113.1 mmol l^−1^ NaCl, 3.0 mmol l^−1^ KCl, 7.5 mmol l^−1^ CaCl_2_·2H_2_O, 20 mmol l^−1^ Tris, 11 mmol l^−1^ glucose, 5 mmol l^−1^ sodium pyruvate, 1 mmol l^−1^ caffeine and 0.1% BSA into the 0.2 ml spermatozoa.

PZM-3 medium: 108 mM NaCl (Sigma, S-5886), 10 mM KCl (Sigma, P-4504), 0.35 mM KH_2_PO_4_ (Sigma, P-5655), 0.40 mM MgSO_4_.7H_2_O (Sigma, M-1880), 25.07 mM NaHCO_3_ (Sigma, S-8875), 0.2 mM sodium pyruvate (Sigma, 4,562) and 2.00 mM Ca(Lactate)_2_. 5H_2_O (Fisher, C100−500), 1.00 mM L-glutamine (Sigma, G-7029), 5.00 mM hypotaurine (Sigma, H-1384), 10 ml l^−1^ NEAA (Sigma, M7145), 20 mg ml^−1^ EAA (Sigma, B-6766), 0.05 mg ml^−1^ gentamicin and 3 mg ml^−1^ BSA (Sigma, A-0281).

### Quantification of 6mA in gDNA by UHPLC–QQQ–MS/MS

Zebrafish embryonic gDNA (20−300 ng) or tissue gDNA (1−2 μg) in 26 μl of nuclease-free H_2_O was denatured at 100 °C for 5 min, chilled on ice for 2 min and digested by 1 μl nuclease P1 (1 U μl^−1^, Wako USA, 145-08221) in 10 mM NH_4_OAc pH 5.3 (adding 3 μl 100 mM NH_4_OAc) at 42 °C overnight. This process was followed with the addition of 3.4 μl NH_4_HCO_3_ (1 M) and 1 μl of phosphodiesterase I from crotalus adamanteus venom (0.001 U, Sigma, P3243-1VL) at 37 °C for 2 h and finally by addition of 1 U of alkaline phosphatase from *E. coli* (Sigma, P5931-500UN) at 37 °C for 2 h. Digested DNA was diluted twofold with nuclease-free H_2_O and was filtered through 0.22 μm filter (Millipore, SLGVR04NL). A portion of 10 μl sample was injected into LC–MS/MS and the nucleosides were separated by reverse-phase UHPLC on a C18 column (Agilent, 927,700-092), with online MS detection using Agilent 6,460 QQQ–MS/MS set to multiple reaction monitoring in positive electrospray ionization mode. Nucleosides were quantified using the nucleoside precursor ion to base ion mass transitions of 266.1−150.0 for 6mA and 252.1−136.0 for A. Quantification of the ratio 6mA/A was performed using the calibration curves obtained from nucleoside standards running at the same time.

### Antibodies

For regular 6mA-IP-seq, the following two rabbit polyclonal anti-6mA antibodies were used: Synaptic Systems (SYSY), 202,003 and Abcam, ab151,230. For immunostaining experiments, rabbit 6mA antibody (SYSY, 202,003) and mouse monoclonal Histone 3 (H3) antibody (Beijing Biodragon Immunotechnologies, B1055F) were used.

### Immunostaining and confocal imaging

For 6mA immunostaining, the zebrafish embryos were fixed in 4% paraformaldehyde at 4 °C overnight. Afterwards, the embryos were dechorionated manually and dehydrated with methanol and rehydrated with PBS, and the embryos were permeabilized for 15 min with PBS containing 1% Triton X-100. The permeabilized embryos were denatured with 2 M HCl for 1 h and then neutralized with 100 mM Tris-HCl (pH 8.5) for 20 min. The embryos were washed and then incubated for 1 h with blocking buffer (1.5% BSA in PBS containing 0.3% Tween and 50 μg μml^−1^ RNase A for RNA digestion). After that, the embryos were incubated with 6mA antibody (1:1,000, SYSY antibody) at 4 °C overnight. Washed thrice with PBS, the embryos were incubated with the secondary antibody at a 1:2,000 dilution (goat anti-rabbit Alexa488, Molecular Probes). Images were acquired using Nikon software on a Nikon Eclipse TI microscopy.

For pig sperm, oocyte and embryo immunostaining, the published protocol was followed^19^. The gametes and embryos were washed with PBS, fixed with 4% paraformaldehyde in PBS for 15 min, and permeabilized with 0.1% Triton X-100 in PBS at room temperature for 15 min. The permeabilized gametes and embryos were incubated in 4 N HCl solution at room temperature for 15 min and followed by neutralization in 100 mM Tris-Cl, pH 8.0 for 30 min. After blocking and incubation overnight with anti-6mA antibody, they were washed and incubated with secondary antibody. Nuclei were stained with DAPI. Immunofluorescence was visualized using confocal laser scanning microscope.

### 6mA dot blot assay

The DNA were denatured by heating at 98 °C for 10 min and spotted on nitrocellulose membranes (GE Healthcare, catalogue number RPN303B). The membrane was baked at 80 °C for 1 h and cross-linked by ultraviolet irradiation, the membrane was blocked in 5% BSA in PBS containing 0.5% Tween 20 (PBST) for 1.5 h at room temperature and then incubated with a 1:10,000 dilution of 6mA antibody (SYSY) overnight at 4 °C. After three washes with PBST, the membrane was incubated with a 1:5,000 dilution of horseradish peroxidase-conjugated anti-rabbit secondary antibody. The membrane was then washed with PBST and treated with enhanced chemiluminescence (ECL).

To estimate the level of 6mA in zebrafish gDNA at different stages, synthetic 6mA-containing DNA oligonucleotide was diluted with unmethylated oligonucleotide to generate standards with a gradient of 6mA content (0.2, 0.1, 0.05, 0.025, 0.0125 and 0.00625%). All the samples and standards were loaded at the same amount. Among the samples we tested, gDNAs at 64-cell, 256-cell, sphere, 13 hpf and 24 hpf stages have 6mA levels of around 0.05, 0.01, 0.004, 0.002 and 0.002%, respectively, which is well consistent with the LC–MS/MS observation.

6mA-containing DNA oligo:

5′-TTGCT(6mA)GGTGGTTGCT(6mA)GGCGGTTGCT(6mA)GGGT-3′

### DNA 6mA-IP-seq and bioinformatic analysis

The published protocol of DNA 6mA-IP-seq was applied^6^. Fifty-basepair single-end sequencing was performed on the Illumina HiSeq2500 platform. Raw sequence reads were mapped to reference genomes (zv10/danRer10 for zebrafish) using Bowtie v1.0.1 (ref. 20), with parameters –M 1, which randomly select one best match if multiple alignments were found. Then MACS^21^ software was used to identify the enriched regions (6mA peaks) by comparing reads from the IP sample with that from the input sample. False discovery rate cutoff was set to 0.01, to select statistically significant peaks. After these peak regions were obtained, the genomic loci were compared with the coordinates of repeat elements downloaded from RepeatMasker database^22^. If more than half length of one peak region overlaps with one annotated repeat element, the peak was annotated as a repeat-originated peak. Gene annotation information was downloaded from UCSC database ( http://hgdownload.soe.ucsc.edu/goldenPath/danRer10/database/). The enrichment of peaks distributed in each genomic region was calculated by HOMER (Hypergeometric Optimization of Motif EnRichment) software^23^.

### Primers for 6mA-containing REs

See Supplementary Fig. 10.

Thy1-F: 5′-ACCTTCACTCCAGCTCCAGA-3′

Thy1-R: 5′-GTGCGGCAGTCTGTCTGATA-3′

Parp4-F: 5′-ATCGTGCTCGTCCTCTCAGT-3′

Parp4-R: 5′-CGGTTTGACCAAAAGCAAAT-3′

Hel1-F: 5′-CAACGACTGCAAGGTGAAGA-3′

Hel1-R: 5′-GGGATTGGAACAACTCCAGA-3′.

### Ethics statement

All animal studies were conducted in strict accordance with the recommendations in the Guide for the Care and Use of Laboratory Animals of the Ministry of Science and Technology of the People's Republic of China. The protocols for zebrafish studies were approved by the Committee on the Ethics of Animal Experiments of the Institute of Zoology, Chinese Academy of Sciences (approval number: IOZ15001). The protocols for pig studies were approved by the Committee on Animal Care and Use and the Committee on the Ethics of Animal Experiments of Zhejiang University.

### Data availability

The data that support the findings of this study are available from the corresponding author upon request.

## Additional information

**Accession codes**: The high-throughput data used in this study are deposited in the NCBI GEO database with accession number GSE76740.

**How to cite this article:** Liu, J. *et al.* Abundant DNA 6mA methylation during early embryogenesis of zebrafish and pig. *Nat. Commun.* **7,** 13052 doi: 10.1038/ncomms13052 (2016).

## Supplementary Material

Supplementary Information Supplementary Figures 1-10 and Supplementary Table 1
