# Supplementary material for: Abundant DNA 6mA methylation during early embryogenesis of zebrafish and pig
Source: Nat Commun. 2016 Oct 7;7:13052. doi: 10.1038/ncomms13052 (PMC5059759; doi:10.1038/ncomms13052)
Supplement: Supplementary Information — Supplementary Figures 1-10 and Supplementary Table 1 [file ncomms13052-s1.pdf]

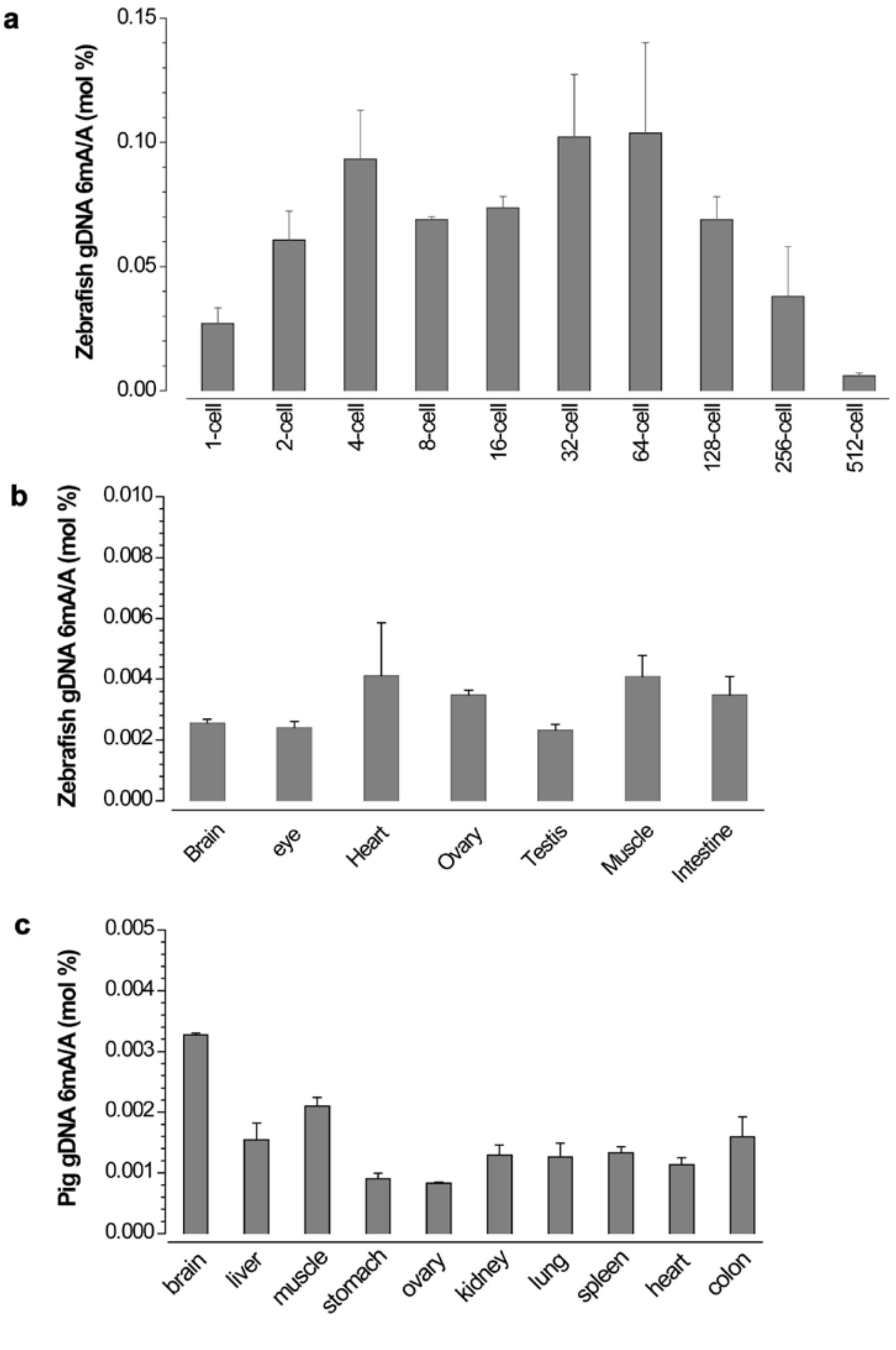

Supplementary Figure 1 | Quantification of 6mA in genomic DNA from zebrafish and pig by UHPLC-QQQ-MS/MS. (**a**) Enlarged view of **Fig. 1a** showing the 6mA abundance of zebrafish embryo stages from 1-cell to 512-cell. The mole ratios of 6mA/A are presented, error bars indicate mean ± s.d. (n = 3). (**b**) 6mA abundance of various tissues from adult zebrafish. Error bars indicate mean ± s.d. (n = 2). (**c**) 6mA abundance of various tissues from adult pig. Error bars indicate mean ± s.d. (n = 2).

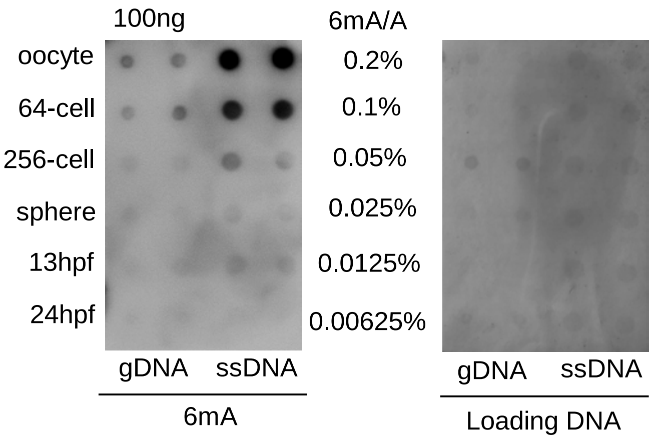

Supplementary Figure 2 | Dot blot assay was performed to measure the 6mA level in zebrafish at different developmental stages as indicated. We have done two replicates for each sample.

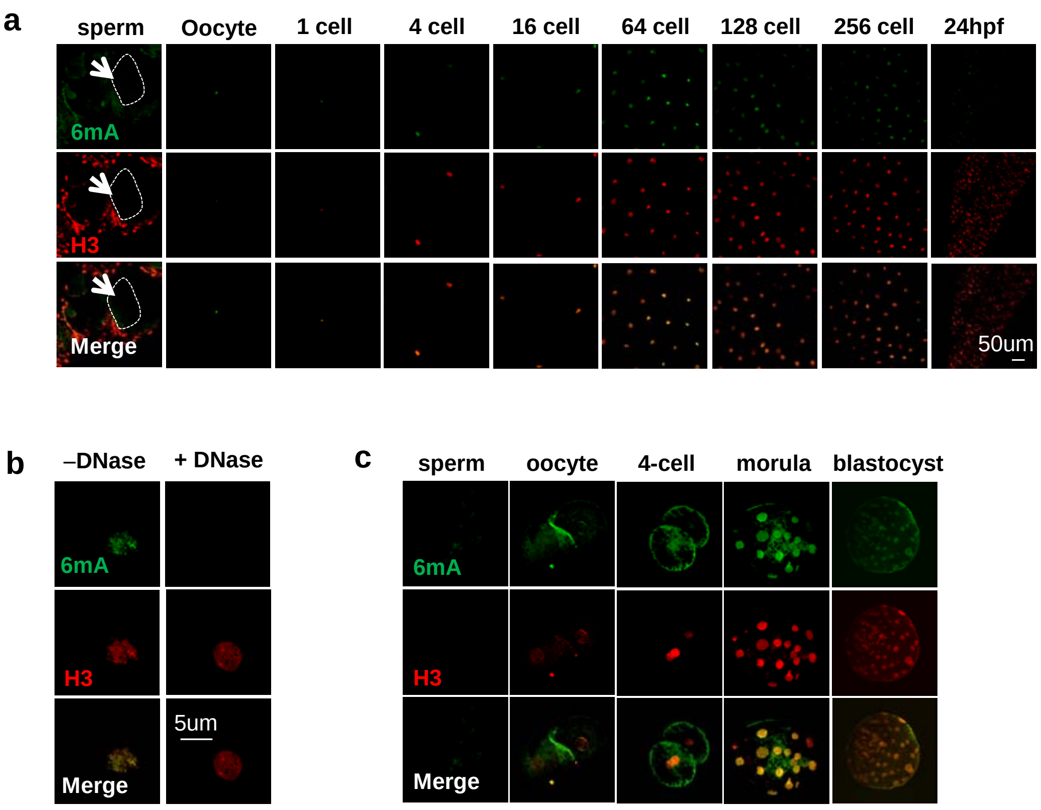

Supplementary Figure 3 | (**a**, **c**) Global view of immunofluorescence images of selected embryo stages of zebrafish (**a**) and pig (**c**) stained by anti-6mA antibody. The dash line-circled area delineates the zebrafish sperm. (**b**) Immunostaining of zebrafish genomic DNA 6mA at 64C stage with and without DNase treatment. Histone 3 (H3) staining was used as counterstain. The scale bars are as indicated.

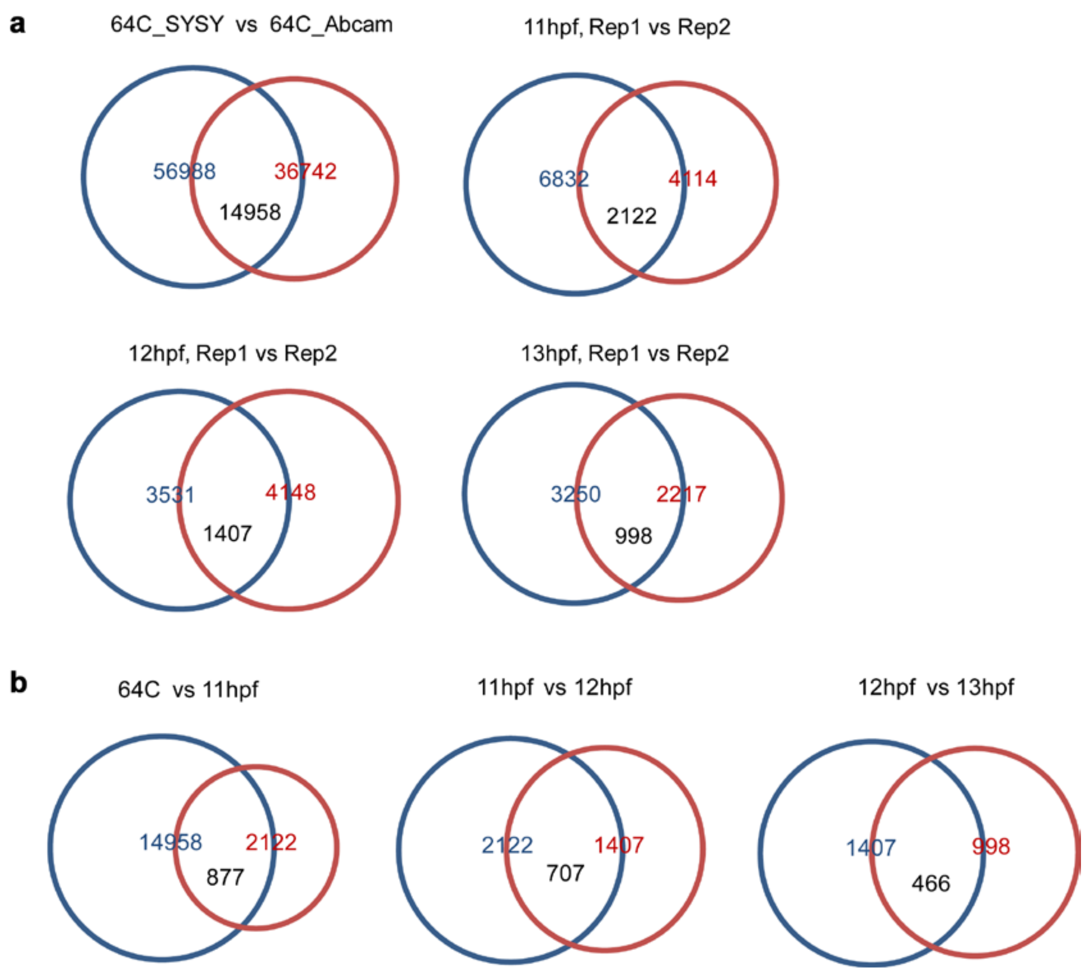

Supplementary Figure 4 | 6mA peaks identified in zebrafish embryo samples. (**a**) Peak overlaps between replicates of 64C, 11hpf, 12hpf, and 13hpf, respectively. For 64C stage, different anti-6mA antibodies SYSY and Abcam were used, while for other stages, the SYSY antibody was used for all replicates. (**b**) Peak overlaps for groups of 64C vs 11hpf, 11hpf vs 12hpf, and 12hpf vs 13hpf. The common peaks of the replicates at each stage were selected for comparison.

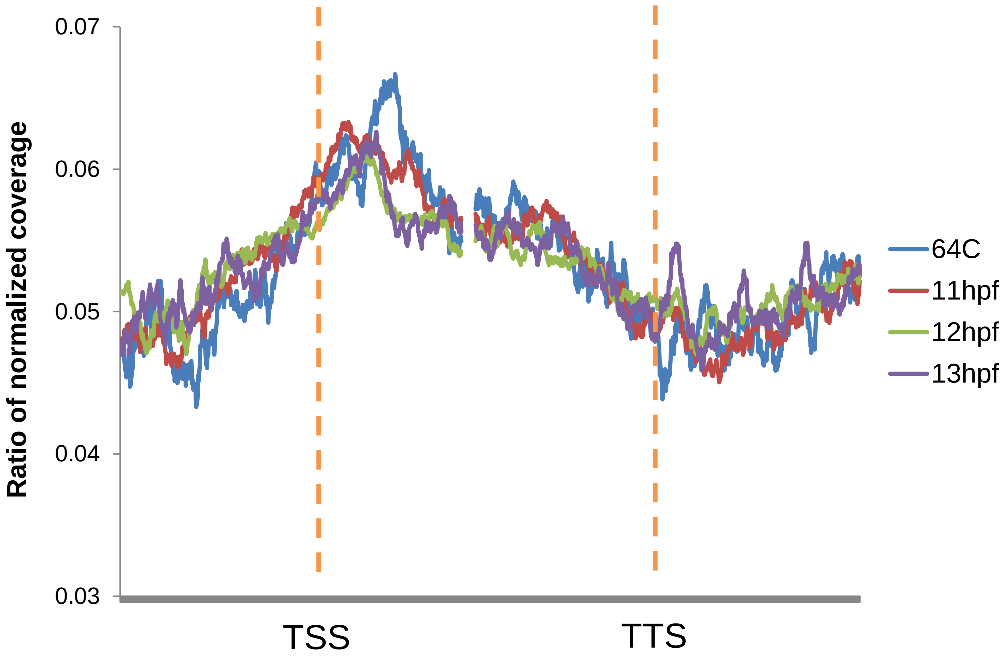

Supplementary Figure 5 | Distribution of 6mA-containing reads around transcription start site (TSS) and transcription termination site (TTS). All the genes are aligned on TSS and TTS. Sequencing reads from upstream to downstream 1k bp of TSS or TTS are accumulated. The 6mA-containing reads (IP) are normalized by dividing the background (input) to eliminate potential sequencing noise. The y-axis is the percentage of normalized reads coverage in each genomic coordinate to the coverage sum across the region.

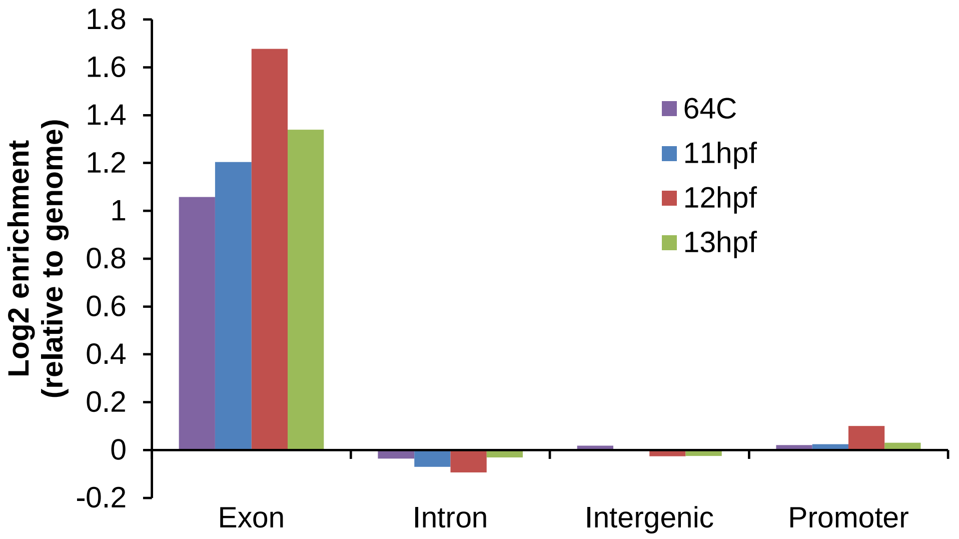

Supplementary Figure 6 | Distribution of 6mA peaks at exon, intron, intergenic, and promoter regions among zebrafish embryonic stages of 64C (64-cell), 11hpf, 12hpf, and 13hpf. The enrichment of peaks distributed in each genomic region was calculated by using the annotatePeaks.pl tool in HOMER software suit.

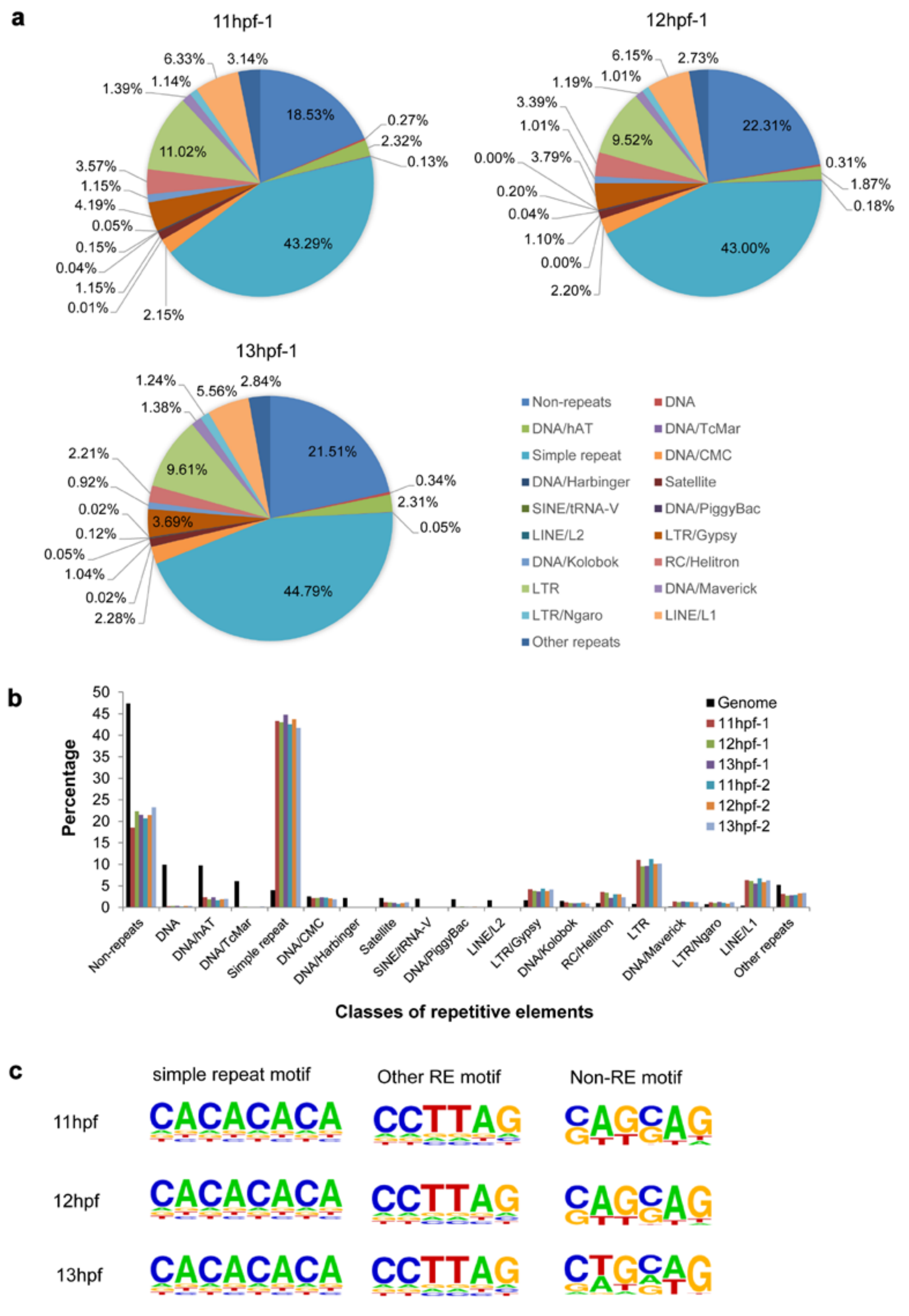

Supplementary Figure 7 | Features of 6mA distribution across the genome of zebrafish at selected embryonic stages. (**a**) Pie chart showing the distribution of 6mA peaks at zebrafish 11hpf, 12hpf, and 13hpf stages. Peaks located in repetitive elements (RE) are classified into subgroups based on RepeatMasker annotation. (**b**) Comparison of 6mA enrichment results for samples of 11hpf, 12hpf and 13hpf, and their replicates. (**c**) Analysis of sequence motifs of 6mA peaks in simple repeat, other RE regions apart from simple repeats, and non-repetitive regions (non-RE). Motifs were searched and generated by using Homer software. The *P* values for simple repeat, RE, and non-RE motifs are all less than 1e-120.

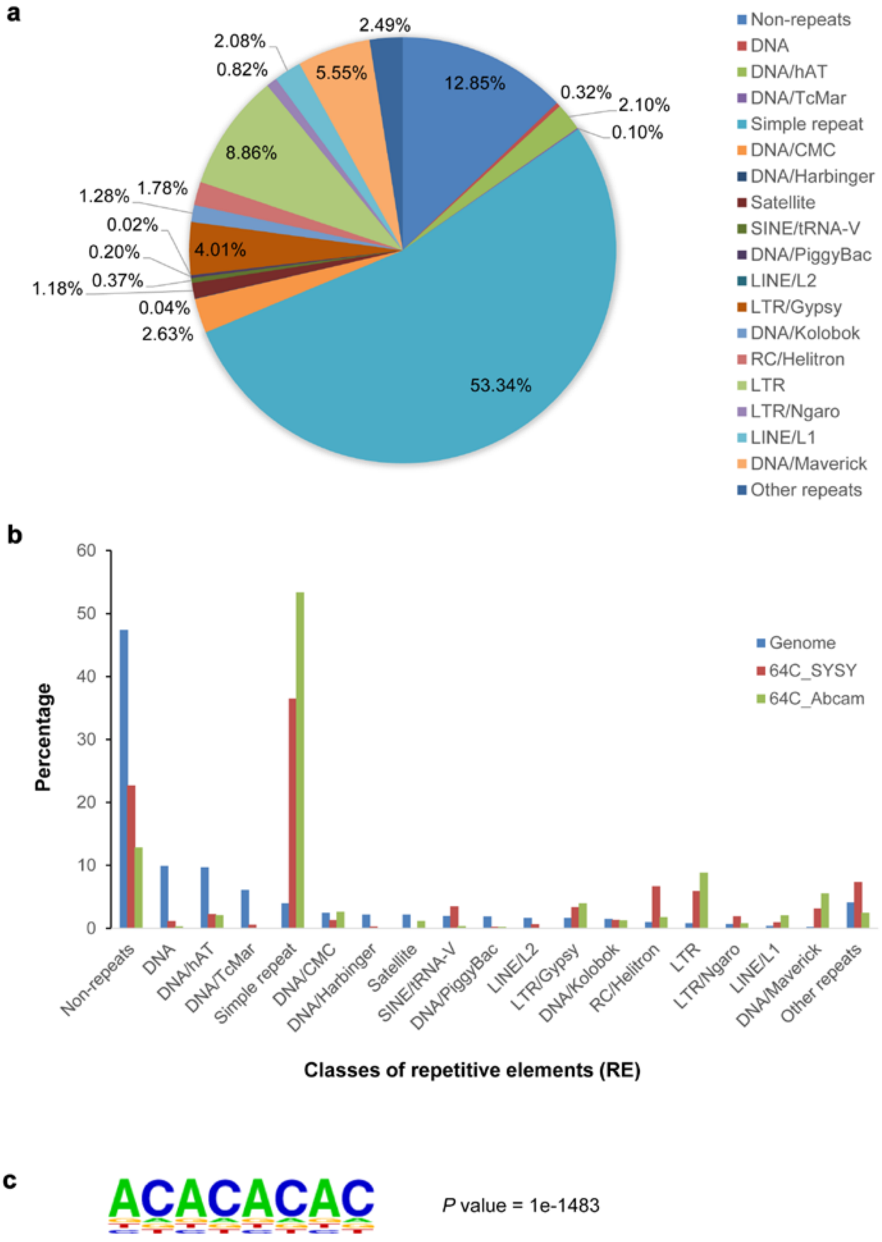

Supplementary Figure 8 | Regular DNA 6mA-IP-seq using a different anti-6mA antibody (Abcam) revealed similar results to that using the SYSY antibody. (**a**) Pie chart showing the distribution of 6mA peaks at 64C stage. Peaks located in repetitive elements are classified into subgroups based on RepeatMasker annotation. (**b**) Comparison of 6mA enrichment results between experiments using SYSY and Abcam anti-6mA antibodies. (**c**) Sequence motif of 6mA peak regions in simple repeat. Motifs were searched and generated by using Homer software.

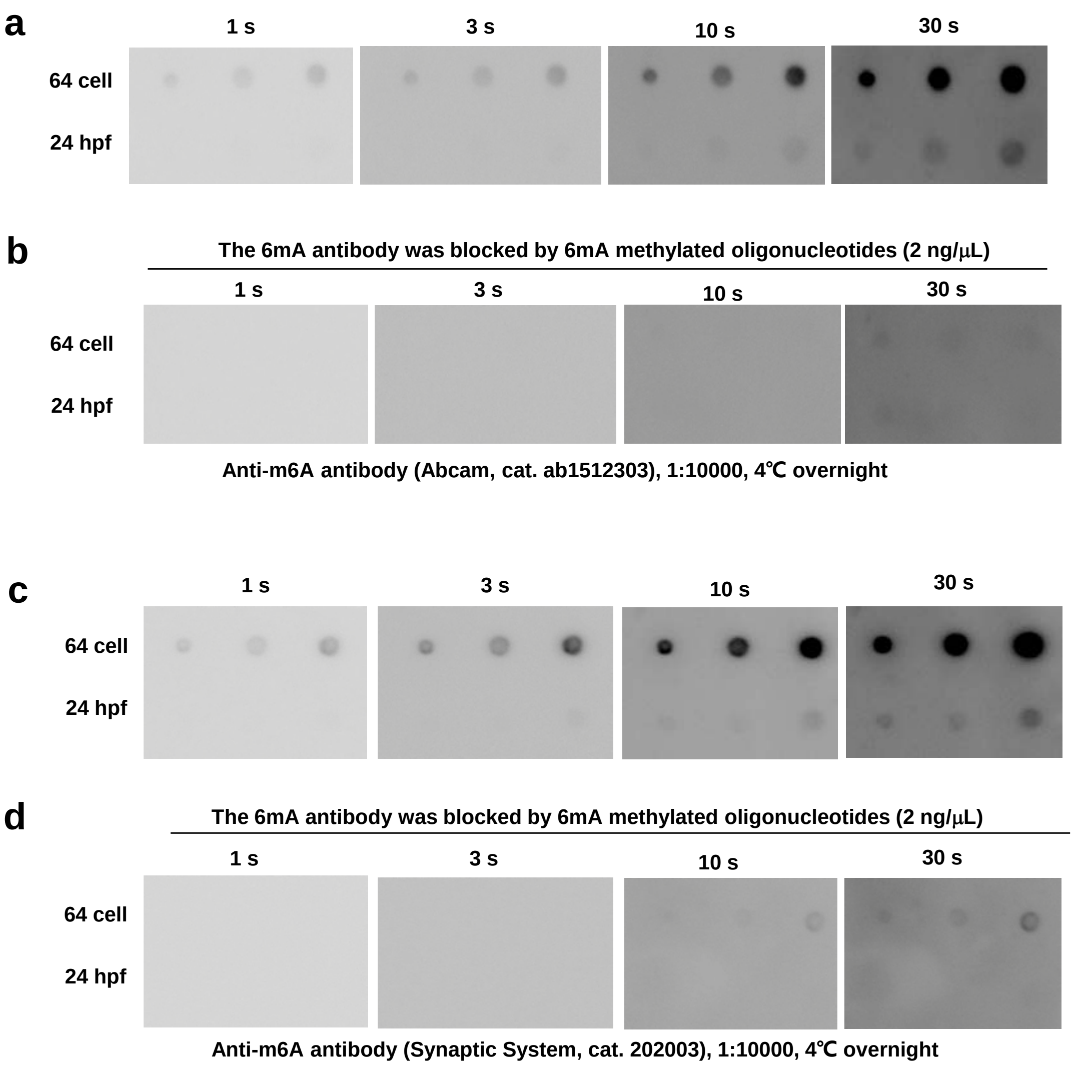

Supplementary Figure 9 | Comparisons of the Abcam and SYSY anti-6mA antibodies using dot blot assay. (**a, c**) Two different stages of zebrafish genomic DNA were subjected to dot blot assays using anti-6mA antibodies from Abcam (cat. ab1512303) and SYSY (synaptic system, cat. 202003). Strong signal was detected at 64C stage, while weak signal was detected at 24hpf stage. (**b, d**) Very weak signal was detected when the 6mA antibody was blocked by the synthesized 6mA-containing oligonucleotide. Different exposure time scales were used. The 6mA-containing oligonucleotide was listed under the subtitle of 6mA dot blot assay in the Methods section.

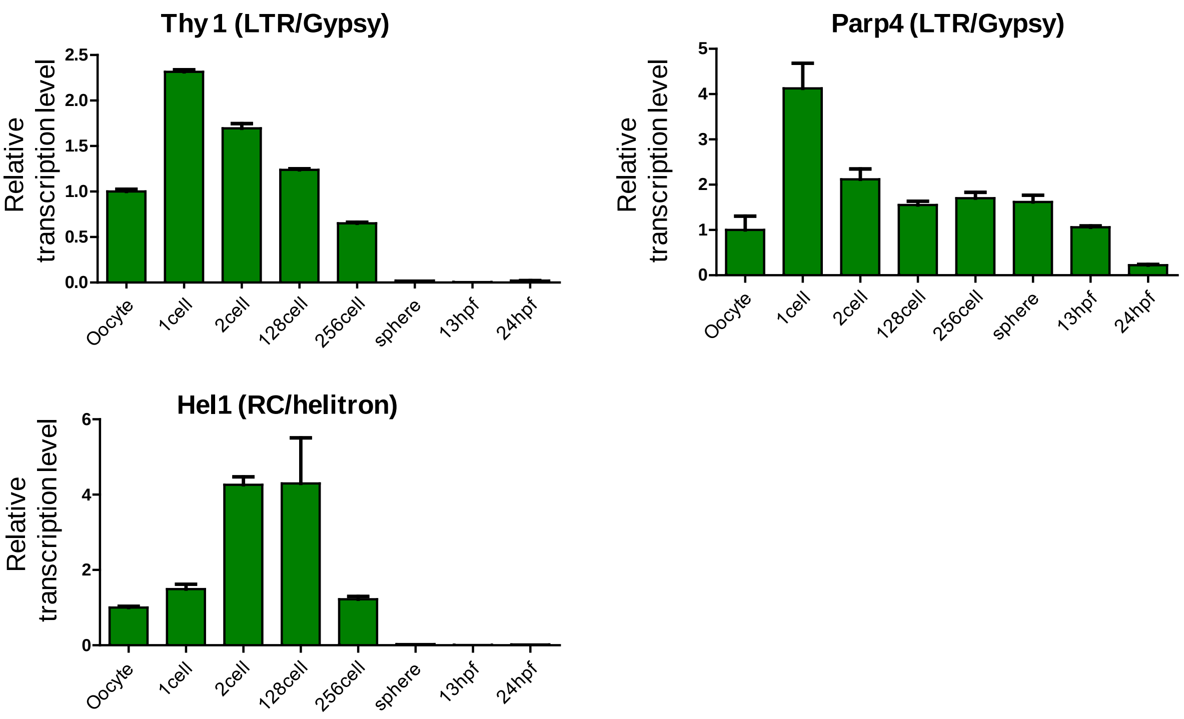

Supplementary Figure 10 | Quantification of expression levels of selected 6mA-enriched repetitive elements at different development stages of zebrafish by RT-qPCR. Thy1: thy-1 cell surface antigen; Parp4: poly(ADP-ribose) polymerase family member 4); Hel1: helitron 1. The descriptions in the brackets denote the classification of the repetitive elements.

Supplementary Table 1. 6mA-IP-seq data summary

Sample name[a]	Total reads	Mappable reads	Mapping ratio
64C input (SYSY)	18308608	14171158	0.774
64C input (Abcam)	56133805	44182666	0.787
11hpf input (SYSY)	25686371	22379024	0.871
12hpf input (SYSY)	24952314	22017605	0.882
13hpf input (SYSY)	29211100	25882420	0.886
64C IP (SYSY)	41595181	29138628	0.701
64C IP (Abcam)	72862385	32786871	0.450
11phf IP (SYSY)	25251395	21905354	0.867
12hpf IP (SYSY)	27466083	23872413	0.869
13hpf IP (SYSY)	24865823	21663812	0.871
11hpf IP (SYSY) replicate	24791367	21584851	0.871
12hpf IP (SYSY) replicate	24906264	21731897	0.873
13hpf IP (SYSY) replicate	29638748	25828903	0.871

[a]The antibody source is indicated in the sample names.
